# An empirical comparative study on the diagnostic index system for techniques and tactics in men's table tennis during the ABS plastic ball Era

**DOI:** 10.3389/fspor.2025.1566717

**Published:** 2025-08-21

**Authors:** Junwei Xu, Qiankun Wang, Ulbossyn Marchibayeva, Nurdybek Bolat, Mukhametkali Sagidolla, Yelshat Kalym, Kairat Aidarkhan, Jan Alam, Ali Zhalel

**Affiliations:** ^1^ Physical Education Institute, Henan University of Economics and Law, Zhengzhou, China; ^2^ Department of Physical Education and Sports, L.N. Gumilyov Eurasian National University, Astana, Kazakhstan; ^3^ Nur-Mubarak University of Islamic Culture of Egypt, Almaty, Kazakhstan; ^4^ School of Competitive Sports, Shanghai University of Sport, Shanghai, China; ^5^ Physical Education and Training Center, King Ali International Trade Limited Liability Company, Astana, Kazakhstan; ^6^ “Shipali” medical center, Astana, Kazakhstan; ^7^ Department of Education, University of Wah, Wah, Pakistan

**Keywords:** MEN, table tennis, technique and tactics, diagnostic index system, segments, New Ball Era

## Abstract

In order to accurately identify the diagnostic index system that can best reflect the technical and tactical performance of table tennis after the implementation of the new ABS material ball, and then more accurately and efficiently diagnose and analyze the technical and tactical ability of players. This paper uses the methods of match observation, mathematical statistics, literature and other methods to carry out an empirical comparative study on the representative segmented diagnostic indicator system constructed by predecessors in the past 20 years. Research suggests that: 1) in the New Ball Era, each segmented diagnostic indicator system has a certain degree of rationality, but in comparison, the five-segment diagnostic indicator system is the most optimal and the most accurate to reveal the technical and tactical performance in the new era. 2) The five segments consist of the serving-attacking segment, receiving-attacking segment, serving-attacking connection segment, receiving-attacking connection segment, and the sustaining segment. 3) The impact of these five segments on the outcome of the match is as follows: The “attacking” segments have the greatest impact on the match outcome, followed by the “connecting” segments, and the “sustaining” segment has the smallest impact. The impact of the receiving and attacking segment on the outcome of the match is higher than that of the serving and attacking segment, and the impact of the serving and attacking connection segment on the outcome of the match is higher than that of the receiving and attacking connection segment. 4) In the New Ball Era, the fifth and sixth strokes of the “connecting” segment have gained prominence, playing a pivotal role for transitioning between offense and defense in the “attacking” segment and the “sustaining” segment. These findings highlight the need for coaches and analysts to adopt the five-segment diagnostic indicator system as a more accurate framework for evaluating performance. Emphasizing training in the receiving-attacking and connecting phases—especially the fifth and sixth strokes, can provide a competitive edge under the dynamics of the New Ball Era. This refined understanding can guide more targeted technical and tactical preparation, better aligning training priorities with the actual determinants of match outcomes.

## 1 Introduction

The practice of Chinese table tennis, which has thrived for over 60 years, has proven that among numerous success factors, technical and tactical diagnostics play an important role of technological support in achieving scientific training and efficiently performing in competitions (1–3). As a classic achievement in the field of quantitative diagnosis of table tennis technique and tactics, the Three-Stage Index Evaluation Method has played a significant role in the Chinese team's multiple victories in World Championships since its inception (4). Due to its high scientific nature, but also effectively reflects the basic characteristics of technical and tactical application in table tennis matches (5, 6). It provides important guidance, diagnosis, and support functions for training and competition (7), and has always been used as the mainstream method of technical and tactical diagnosis, continuing to be used until today (4, 8, 9).

Since the beginning of the new century, frequent rule changes have led to constant development and changes in the table tennis technical and tactical system, especially after the International Table Tennis Federation (ITTF) replaced the celluloid table tennis ball with the ABS(Acrylonitrile Butadiene Styrene) plastic material table tennis balls (referred to as “new balls” hereinafter) since 2017, the spin of the ball has been further reduced while the elasticity has increased slightly (10). This has led to further development and widespread application of techniques such as loop drive within close-table, counter drive, and quick connection (11, 12). This has not only brought significant changes to the table tennis technical and tactical system (13–19), but has also presented numerous problems for the application of the Three-Stage Index Evaluation Method (9, 12, 13, 20–22).

Over the past 20 years, to better adapt the Three-Stage Index Evaluation Method to the development of table tennis techniques and tactics, the academic community has continuously revised and improved the diagnostic index system of this method. This method has gradually progressed from “Elementary Stage” to “New Three-Stage” (13, 23), “Four Stages” (3 24, 25), “Five Stages” (26), “Six Stages” (27), and “Dynamic Three-Stages” (9, 12). However, as the majority of these ways of segment divisions are constructed based on subjective experience and then tested their construction effect with a small number of match cases, there is a severe lack of highly persuasive empirical constructions based on representative large samples, this not only partially disrupts people's accurate understanding of the characteristics and laws of table tennis competition but also brings confusion to subsequent scholars in choosing and applying the “paradigm” of technical and tactical diagnosis (4, 12, 18, 28). Meanwhile, because most quantitative analysis studies on table tennis techniques and tactics are currently based on extensions and developments of various segmented diagnostic index system frameworks centered around the classic “Three Stages” (9, 29, 30). If a diagnostic index system that best fits the performance of table tennis techniques and tactics in the New Ball Era cannot be objectively and accurately identified, it will inevitably affect the overall effectiveness and benefits of the analysis of Chinese men's table tennis technical and tactical diagnosis.

Furthermore, although there are also some studies that have utilized the development and usage of computer software to conduct big data mining analysis on winning table tennis techniques and tactics, such as sequence pattern mining (31, 32), association rules (32), ant colony algorithm (33), decision tree algorithm (34, 35) etc., the process of such research is complex, the results are abstract, and practical applications often have many limitations (4, 36–39). Moreover, some studies do not select effective tactics starting from serving and receiving serves. This cherry-picking style of mining often lacks practical application value, and is far less simple, practical, and efficient than segmental diagnostic methods centered around the Three-Stage Index Evaluation Method.

Therefore, the author intends to base the analysis on the technical and tactical performance of male athletes in high-stage matches (finals, semi-finals, quarter-finals) of the major events after the implementation of the new ball. Using structural equation modeling as a tool, we conduct a large sample empirical comparative study on the segmented diagnostic index system, which is currently representative in academic circles. In order to identify the most suitable diagnostic index system for table tennis technical and tactical performance in the New Ball Era, we aim to fully leverage the diagnostic, predictive, and guiding functions of the diagnostic index system. This will provide valuable references for the scientific training and improvement of preparing and joining the competition effectiveness for the Chinese men's team.

## 2 Methods

In this study, the research object considered is an empirical comparison of diagnostic index system for men's table tennis technical and tactical analysis in the New Ball Era.

### 2.1 Literature review method

Based on the research needs, literature on table tennis technical and tactical diagnostic methods since the establishment of the 1988 Three-Stage Index Assessment Method was selected and sorted through the China National Knowledge Infrastructure (CNKI) to comprehensively grasp the latest research results and evolutionary trends related to quantitative diagnostic methods of table tennis technics and tactics.

### 2.2 Expert interview method

To determine more representative diagnostic index systems and understand the characteristics of various segmented diagnostic systems, interviews were conducted with four leading domestic table tennis experts. These experts (including one senior professor, two national-level coaches, and one technical and tactical specialist from the Chinese table tennis team) had an average age of 44. Through three rounds of iterative interviews, all four experts ultimately reached a consensus.

### 2.3 Match observation method

#### 2.3.1 Selection of observation subjects

In order to enhance the representativeness of the study subjects and to avoid situations where statistical data cannot reflect the true abilities of athletes due to significant differences in their competitive strength (40–42), the men's singles finals, semi-finals, and quarter-finals of the 12 major international table tennis tournaments recognized by the world after the implementation of the new ball are selected as the observation objects for the competition (Table 1). The total number of matches observed is 168, with a total of 17,070 game rounds. All athletes employ offensive play.

**Table 1:** Number of matches in each event in 2017—2024.

Types of sports competition	Finals	Semi-finals	Quarter-finals
The 2017 World Table Tennis Championships	1 × 2	2 × 2	4 × 2
The 2017 Table Tennis World Cup	1 × 2	2 × 2	4 × 2
The 2018 Table Tennis World Cup	1 × 2	2 × 2	4 × 2
The 2019 World Table Tennis Championships	1 × 2	2 × 2	4 × 2
The 2019Table Tennis World Cup	1 × 2	2 × 2	4 × 2
The 2020 Table Tennis World Cup	1 × 2	2 × 2	4 × 2
The 2020 Olympic Games	1 × 2	2 × 2	4 × 2
The 2021 World Table Tennis Championships	1 × 2	2 × 2	4 × 2
The 2022 WTT Grand Slam tournament	1 × 2	2 × 2	4 × 2
The 2023 WTT Grand Slam tournament	1 × 2	2 × 2	4 × 2
The 2023 World Table Tennis Championships	1 × 2	2 × 2	4 × 2
The 2024 WTT Grand Slam tournament	1 × 2	2 × 2	4 × 2
Total	24	48	96

Due to the need to separately record data for both players in each match, the sample size is twice the actual number of matches.

#### 2.3.2 Observation nodes and methods

Based on the composition of various segmented diagnostic index systems, observation nodes are established, with the last board scored (or lost) by each player in each game round serving as the observation basis. This was done independently by each of the 2 master's degrees in table tennis, and then the data were compared on a game-by-game, indicator-by-indicator basis, and when the data were inconsistent, the authors and observers reobserved and corrected for that data until all data were accurate and consistent.

### 2.4 Mathematical statistics

#### 2.4.1 Statistical indicators and evaluation parameters

The statistical indicators are the paragraphs of the segmented diagnostic indicator system involved in empirical comparison (Table 2), and the evaluation parameters are the scoring rate, utilization rate of each segment, and the probability of winning each match. The specific calculation methods are as follows:

$$\begin{array}{c} \mathrm{Scoring}\;\mathrm{rate}=[\mathrm{Segment}\;\mathrm{scores}\;/\;\mathrm{Segment}\;(\mathrm{scores}+\mathrm{losses})]\times 100\text{\%}\\ \mathrm{Utilization}\;\mathrm{rate}=[\mathrm{Segment}\;(\mathrm{scores}+\mathrm{losses})\;/\;\mathrm{Match}\;(\mathrm{scores}+\mathrm{losses})]\times 100\text{\%}\\ \mathrm{Winning}\;\mathrm{probability}=[\mathrm{Match}\;\mathrm{scores}\;/\;\mathrm{Match}\;(\mathrm{scores}+\mathrm{losses})]\times 100\text{\%} \end{array}$$

**Table 2:** Table tennis segmented diagnostic Index system of technique and tactics (1992.1——2024.1).

Category	Scoreboard of points earned and lost in each segment	Initial application time	First-time used by the author	№ of uses	Percentage (%)
3 segments	①serving and attacking segment:1,3 board; receiving and attacking segment: 2,4 board; sustaining segment: the 5th board and subsequent boards.	1988	Wu Huanqun	65	63.7%
	②serving and attacking segment:1,3,5 board; receiving and attacking segment: 2,4 board; sustaining segment: the 6th board and subsequent boards.	2004	Zhang Xiaopeng	6	5.9%
	③serving and attacking segment:1,3,5 board; receiving and attacking segment 2,4,6 board; sustaining segment: the 7th board and subsequent boards.	2005	Zhang Xiaoting	2	2%
	④dynamic 3 segment: serving and attacking segment:1,3,5 board; receiving and attacking segment: 2,4 board; sustaining segment: the 4th board and subsequent boards. Among them, the 4th and 5th boards are dynamically divided according to certain standards.	2014	Wu Fei	3	2.9%
4 segments	⑤serving and attacking segment:1,3 board; receiving and attacking segment: 2,4 board; serving rotation sustaining segment: the 5th board and subsequent odd-numbered boards; receiving and serving rotation sustaining segment: the 6th board and subsequent even-numbered boards.	2005	Dong Yang	11	10.8%
	⑥serving and attacking segment:1,3,5 board; receiving and attacking segment: 2,4 board; serving rotation sustaining segment: the 5th board and subsequent odd-numbered boards; receiving and serving rotation sustaining segment: the 6th board and subsequent even-numbered boards.(the scores of the 5th board are included in the sustaining segment, the lost points are attributed to the serving and attacking segment.)	2014	Yáng Qīng	7	6.9%
	⑦serving and attacking segment, receiving and attacking segment, sustaining segment Ⅰ,sustaining segmentⅡ.The connection after the spin of the first board serves as the beginning of the sustain, and each segment is not limited by the number of boards.	2022	Cheng Lin	1	1%
*5 segments*	⑧serving and attacking segment:1,3 board; receiving and attacking segment: 2,4 board; serving rotation connection segment: the 5th board; receiving and serving rotation connection segment: the 6th board; sustaining segment: the 7th board and subsequent boards.	*2015*	*Jiang Jinjun*	*3*	*2.9%*
6 segments	⑨serving and attacking segment:1,3 board; receiving and attacking segment: 2,4 board; serving and attacking connection segment: the 5th board; receiving and attacking connection segment: the 6th board; serving rotation sustaining segment: the 7th board and subsequent odd-numbered	2010	Tang Jianjun	4	3.9%

“Stroke number” refers to the sequential position within a rally at which the point-winning or point-losing stroke was struck (or should have been struck, theoretically), counting from the first stroke of the rally.

#### 2.4.2 Data analysis methods

Given the superiority of Structural Equation Modeling (SEM) in both model validation (capable of simultaneously handling relationships among multiple observed and latent variables) and model comparison, the Jarque–Bera test was first employed to assess the normality of the observed variables (i.e., the evaluation parameters for each indicator). Threshold values for the kurtosis coefficient and skewness coefficient of the observed variables were set at 5.0 and 2.0, respectively. If the data met the requirements for normal distribution, the various incorporated segmented diagnostic index system structural models were then tested and analyzed using AMOS 26.0 statistical software, with the Maximum Likelihood method selected for estimation. Finally, an independent-samples *T*-test was used to empirically validate the optimal diagnostic index system identified in the previous step.

### 2.5 Research pathway design

The research pathway unfolds through six sequentially integrated phases, beginning with problem formulation, specifically determining how to identify diagnostic index systems that best capture table tennis technical and tactical performance in the New Ball Era. This proceeds to methodology selection, primarily employing match video analysis and structural equation modeling (SEM). Subsequent stages advance through diagnostic system screening via a three-step process (literature-based, logical, and expert screening), followed by data collection, processing, and verification. The pathway then progresses to structural model specification, estimation, quality assessment, and comparative analysis, culminating in discussion of findings and evidence-based conclusion derivation.

## 3 Research findings and analysis

### 3.1 Screen of diagnostic index system

Professor Xing Wenhua has pointed out that the construction of indicator systems for research in technical and tactical diagnostic evaluation should follow two principles. First, the construction of the indicator system should meet the requirements of logical analysis, that is, the connotations of each indicator should be mutually independent and mutually exclusive. Second, the construction of the indicator system or the selection of indicators should be determined by expert experience (32). To ensure that the selected diagnostic indicator system has strong representativeness and comparability, this study adopts a screening method combining literature screening, logical screening, and expert screening.

#### 3.1.1 Literature screening

In 1988, the establishment of the three-stage indicator evaluation method marked the basic formation of quantitative analysis methods for technical and tactical aspects of Chinese table tennis. Using the principle of holistic and logical construction of this method-“dividing the athlete's overall competition ability into segments from the overall level, where the overall competition ability equals the sum of the abilities in each segment,” as the screening principle for the diagnostic indicator system. Through comprehensive screening and sorting of the “Table Tennis Technical and Tactical Analysis and Diagnosis” articles published in Chinese core journals from January 1992 to January 2024 in China, it was found that: Firstly, the finding is that there are four categories and nine species of segmented diagnostic indicator systems created and adopted by predecessors, where “four categories” refer to the three-stage, four-stage, five-stage, and six-stage diagnostic indicator systems.

The “9 species” refer to the diagnostic indicator systems further classified based on the different gain or loss score strokes included in each segment on the basis of the “4 categories” (refer to Table 2). In terms of application, the utilization proportion of the three-stage diagnostic indicator system with the classic three-stage indicator method at its core ranks first, with a utilization rate of 74.5%. The utilization rate of the four- stage diagnostic indicator system ranks second, accounting for 18.6%. The utilization rates of the five-stage and six-stage diagnostic indicator systems are the lowest, at 2.9% and 3.9% respectively. Secondly, in terms of “first adoption time”, apart from the classic three-stage diagnostic indicator system, the “first adoption time” of the other eight diagnostic indicator systems is all distributed after the year 2000.

**Figure 1:**
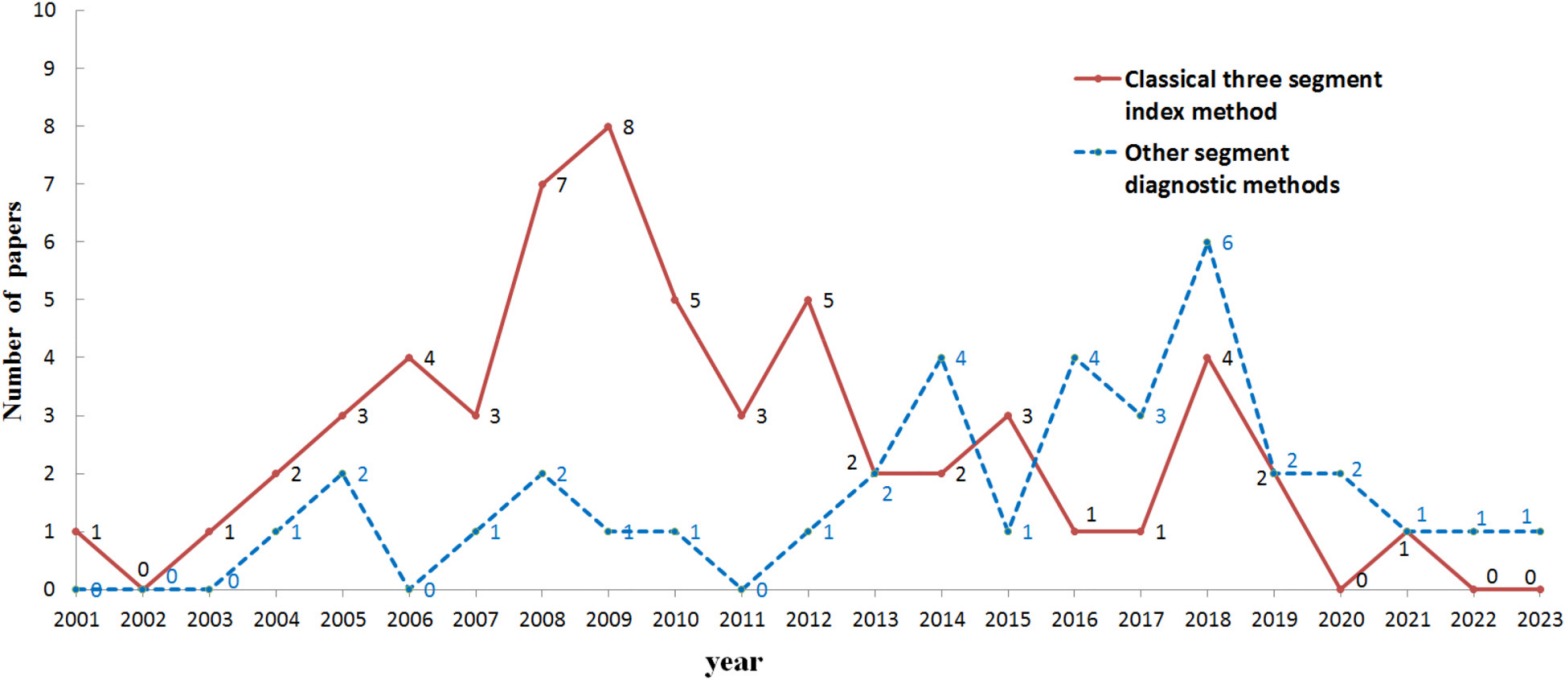
The number of papers using the three-stage index method and other segmented diagnostic methods in each year since 2000.

Furthermore, looking at the application of various segmented diagnostic methods after the year 2000 (as shown in Figure 1), it is observed that the application of the classic three-stage indicator method peaked around the time of the 2008 Beijing Olympics, and has since shown a significant overall downward trend. This is mainly due to the frequent changes in the rules of table tennis after 2000 and the continuous development of technology. This situation also indicates to some extent that rule changes and the rapid development of technology have indeed posed significant challenges to the application of the classic three-stage indicator assessment method. Therefore, it is particularly urgent and necessary to select from various segmented diagnostic indicator systems the one that best fits the current performance of table tennis technique and tactics.

#### 3.1.2 Logical screening

In literature screening, some scholars have established itemized diagnostic indicator systems based on specific research purposes and focusing on key tactical indicators (see Table 3). However, upon observing and analysing the specific indicator composition, it is found that: First, the logical structure of its indicator system is not rigorous enough, violating the logical requirement that “the parent item equals the sum of each sub-item, and each sub-item is independent and mutually exclusive” when categorizing objects. Secondly, the scoring rate and utilization rate are the most basic evaluation parameters of technical and tactical indicators. However, the various indicators (or partial indicators) of these four itemized diagnostic indicator systems only consider the scoring rate and neglect the utilization rate, which will inevitably reduce the integrity and evaluative effectiveness of the diagnostic indicator system to some extent. Based on the above two points, these four itemized diagnostic indicator systems were not included in the sequence of segmented diagnostic indicator systems mentioned above.

**Table 3:** List of Sub-item diagnostic Index system.

Name	The composition of each diagnostic index system	Publication time	Author
Ten-Point Index Method	serving scoring rate; serving-attacking utilization rate, scoring rate, hit rate; serving counterattacking utilization rate; receiving-attacking utilization rate, hit rate, scoring rate; controlled connection attacking utilization rate, sustaining scoring rate	1998	Li Jinliang
Eleven-Point Index Method	first, second, third, fourth shot scoring rate, second, third, fourth shot lost scoring rate, scoring rate and lost scoring rate of sustaining segment I, segment II	2016	Kong Linghui
Seven-Point Index Method	the scoring rate of serving, serving-attacking, serving controlling, receiving serving, receiving-serving continuous attack, receiving serving control and attack, sustaining index etc.	2017	Zhao Xiying
Six-Point Index Method	the scoring rate of serving, serving-attacking, receiving serving, serving-receiving attack, receiving serving control and attack and active sustained index etc	2018	Zhao Xiying

#### 3.1.3 Expert screening

The results of the expert interviews on the nine diagnostic indicator systems screened in the two ways mentioned above show that, firstly, in response to individual scholars' practice of “rigidly” dividing the 4th stroke and the 5th stroke into different segments (3, 9, 12). Most experts agree that each score stroke or lost stroke is an independent competitive unit, and that when analysing the technical and tactical statistics of individual matches for special diagnostic purposes, it is not unreasonable to “rigidly” divide a score stroke or lost stroke into different segments.

Wu Fei is to better match Wang Hao's technical style, however, in order to construct a common system of indicators that can diagnose the playing ability of the majority of athletes and that can accurately fit and reflect the current stage of technical and tactical performance in table tennis, it is not reasonable to “rigidly” divide the score stroke or lost stroke into different segments (13, 19, 27). Secondly, experts also believe that the “Type④” segmented diagnostic index system is overly detailed in its classification, overly complicated in its statistical process, and has limited applicability (9, 12). The “Type⑦” segmented diagnostic index system breaks through the limitation of the number of score stroke or lost stroke and relies solely on “the connection after the first stroke's topspin serve as the dividing line for the sustaining segment” (16). This will inevitably lead to inconsistent connotations within the same segment data. Therefore, this study will exclude these two diagnostic index systems and only retain the “Type⑥” of diagnostic index system, which defines such “division” in a simplified manner, for reference-based empirical comparative research.

Finally, based on literature screening, logical screening, and expert screening, seven diagnostic index systems, namely “Type①”, “Type②”, “Type③”, “Type⑤”, “Type⑥”, “Type⑧”, and “Type⑨”, were selected for empirical comparative analysis.

### 3.2 Empirical comparison and analysis of diagnostic index systems

The empirical comparison of diagnostic index systems mainly consists of four steps: the first is model construction, that is, constructing a structural model that can truly reflect the segment structure of each segmented diagnostic index system and its relationship with the match results; The second step is sample data inspection, which involves checking whether the sample data meets the requirements for structural equation model analysis. The third step is model estimation, which involves initiating the confirmatory factor analysis procedure to test whether the model can be identified. The fourth step is the quality inspection and comparison of the model, which involves examining and comparing the overall fit indices and the internal structural fit indices of the model.

#### 3.2.1 Model construction

**Figure 2:**
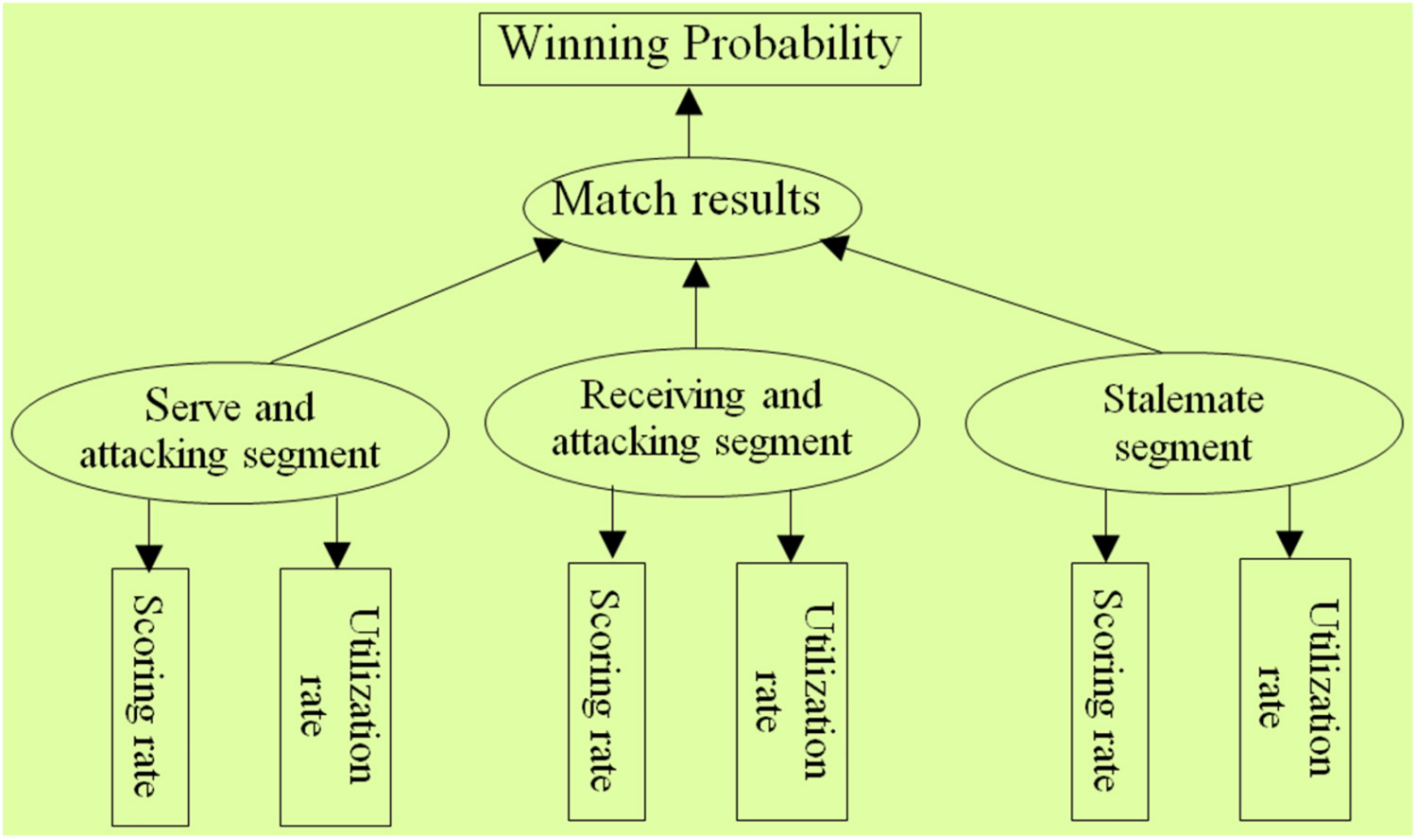
Model of classic three stage diagnostic Index architecture.

As can be seen from the above analysis, the 6 segmented diagnostic index systems such as “Type②”, “Type③”, “Type⑤”, “Type⑥”, “Type⑧”, and “Type⑨” are all based on the segment division principle of the classic three-stage index method “Type ①”. Starting from the overall view of athletic ability, they divide the overall athletic ability of athletes into independent yet interconnected segments (43). Then, scoring rate and utilization rate are used as the basic evaluation parameters for segments. According to training theory, the athlete's own athletic ability is the fundamental internal cause determining the match result. Similarly, the tactical abilities demonstrated by athletes in each segment are also internal factors determining the match result. Based on the above segment composition structure and evaluation parameters of the 7 diagnostic index systems, as well as the inherent relationship between athletes' athletic ability and match results, the structural models of the segmented diagnostic index systems are constructed as follows: (1) Take the segments contained in each diagnostic index system as independent variables (or latent external variables), and take the match results as dependent variables (or latent internal variables). (2) Take scoring rate and utilization rate parameters as observed variables reflecting the tactical abilities of each segment. (3) Take the winning probability of each match as an observed variable reflecting the match results (specific examples can be seen in the structural model diagram of the classic three-stage diagnostic index system) as shown in Figure 2.

#### 3.2.2 Sample data verification

Before using structural equation modeling techniques, it is necessary to verify whether the sample data meets four criteria. These criteria are as follows: (1) each observed variable is a continuous variable; (2) there are at least two observed variables for each latent variable; (3) the data distribution of each observed variable should be normal; (4) an appropriate sample size is available.

a: The observed variables in this paper (scoring rate, utilization rate) are all continuous variables, with two observed variables for each latent variable, thus meeting the requirements of the first two tests.
b: Using the Jarque–Bera test to conduct a normality test on the observed variables in the structural model of the 7 segmented diagnostic index systems constructed above. The test results show that the highest absolute values of kurtosis and skewness coefficients for all observed variables in each structural model are 4.383 and 1.649, respectively, which are both smaller than the reference values of 5.0 and 2.0 for these coefficients, thus basically meeting the requirement of normal distribution.
c: Rigdon (2005) suggested that in structural equation modeling analysis, if the variables under study follow a normal distribution, having 10 samples per observed variable is sufficient. In this study, the 9th diagnostic index system has the most observed variables, with 12 in total. With a sample size of 168, the requirements for sample size are met.

#### 3.2.3 Model estimation

**Figure 3:**
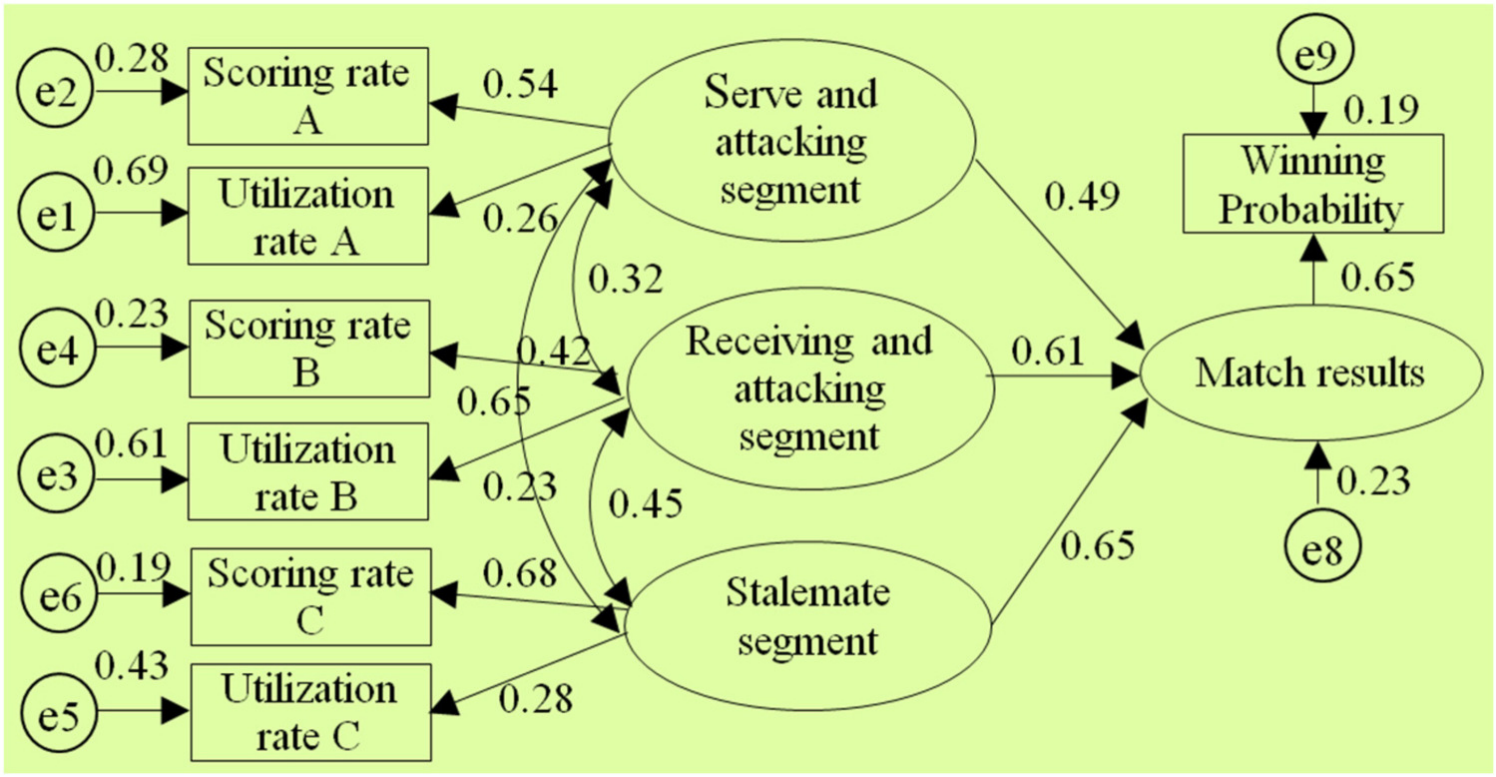
Diagram of the “Type①” diagnostic indicator system architecture model.

**Figure 4:**
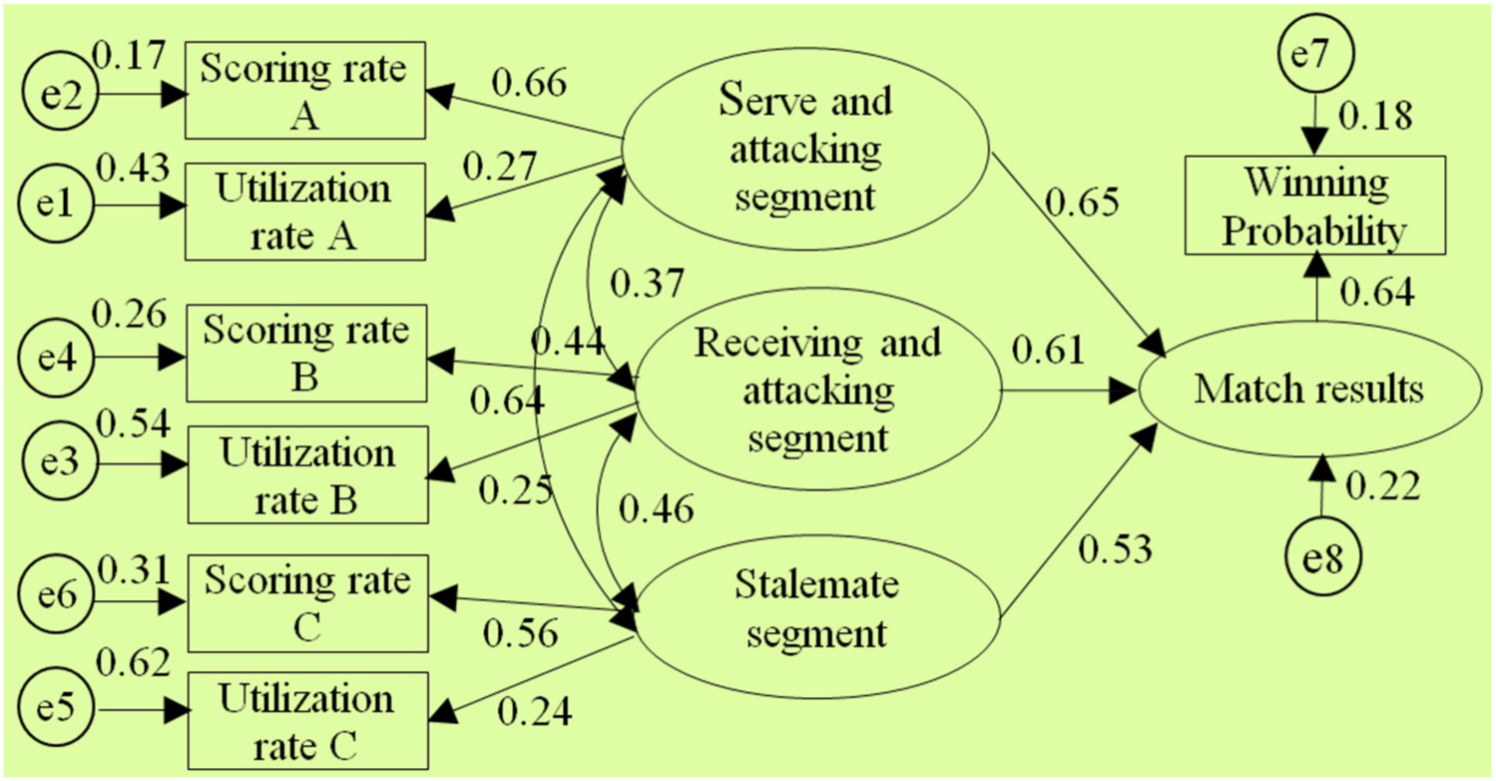
Diagram of the “Type②” diagnostic indicator system architecture model.

**Figure 5:**
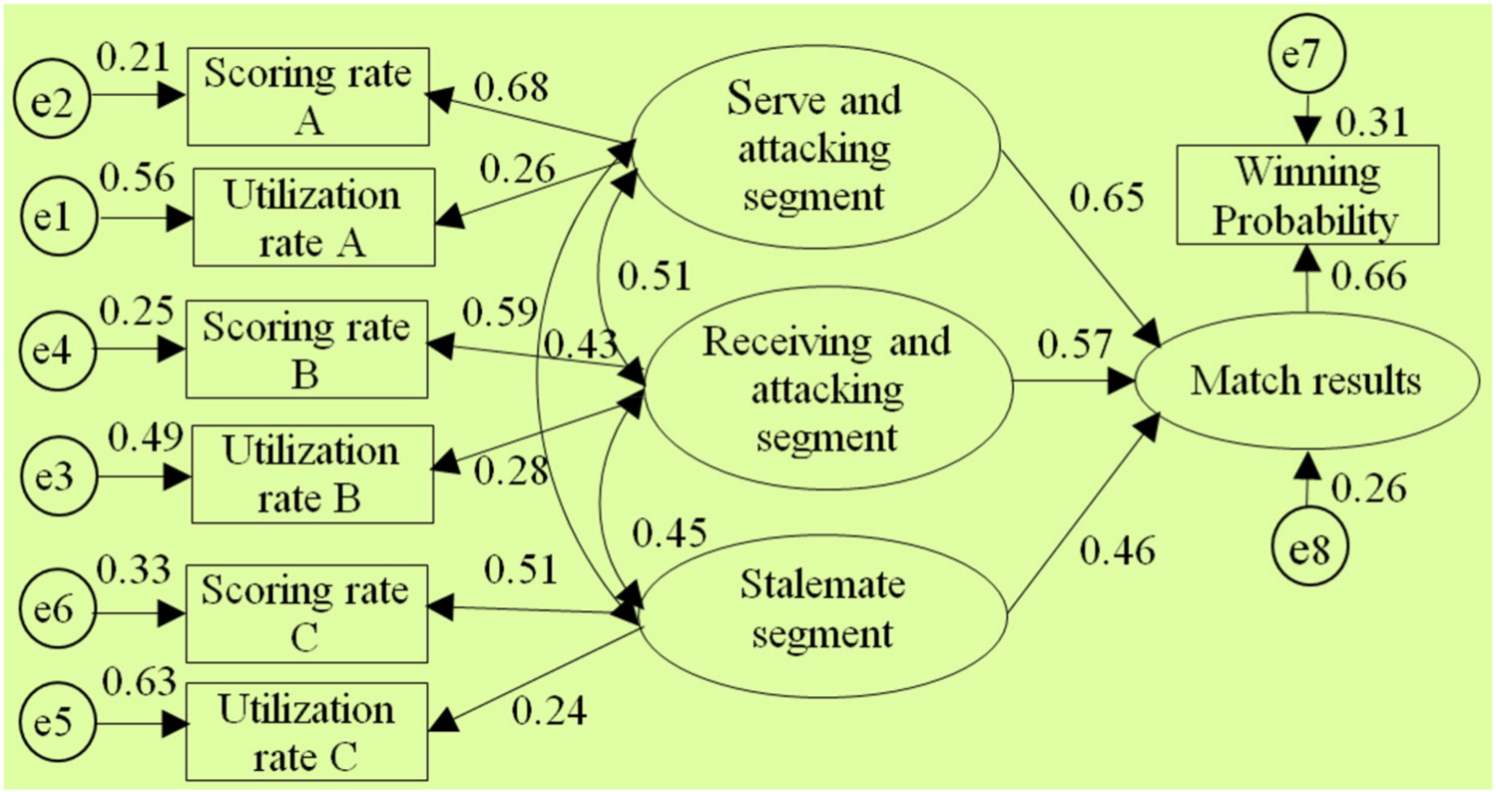
Diagram of the “Type③” diagnostic indicator system architecture model.

**Figure 6:**
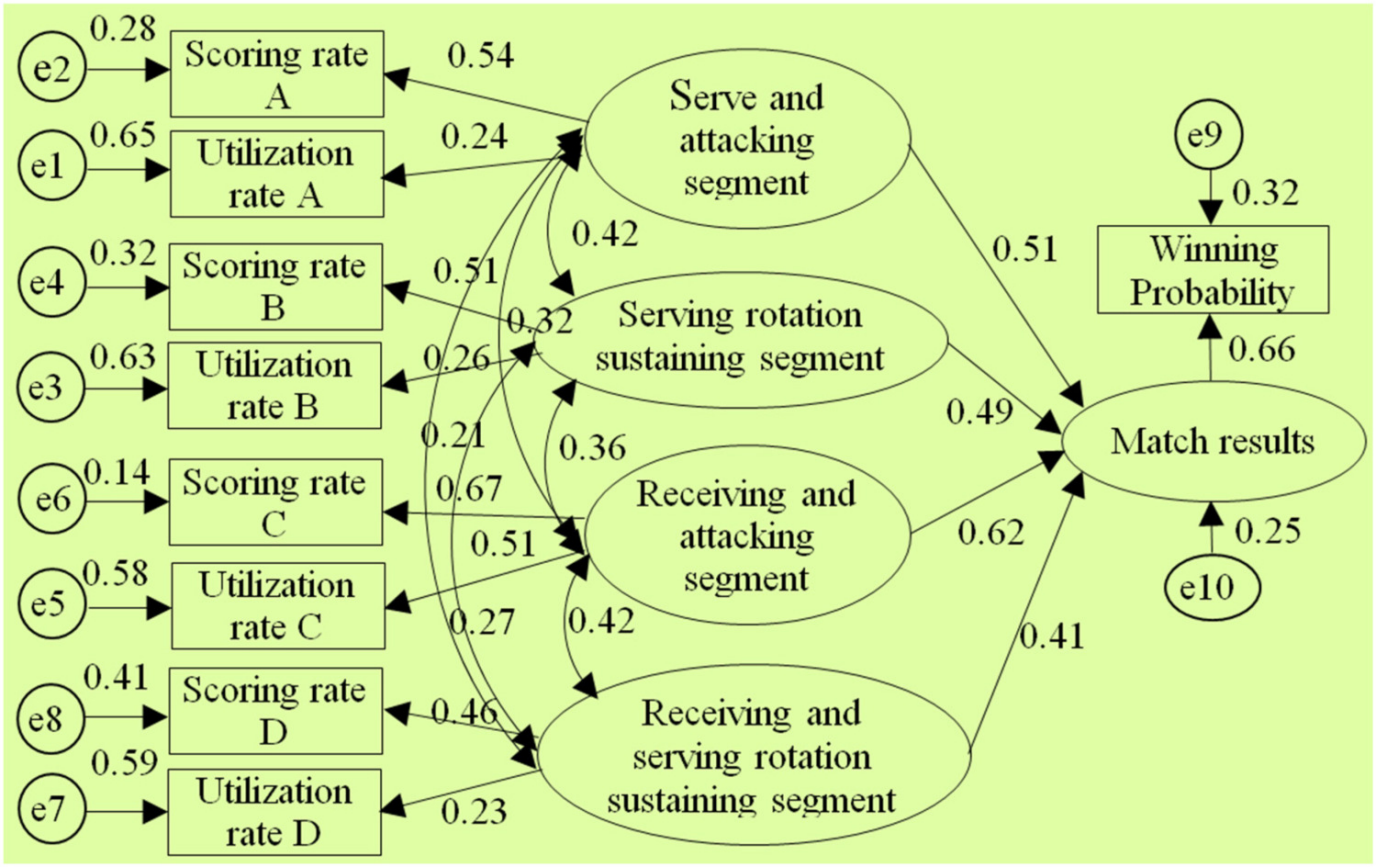
Diagram of the “Type⑤” diagnostic indicator system architecture model.

**Figure 7:**
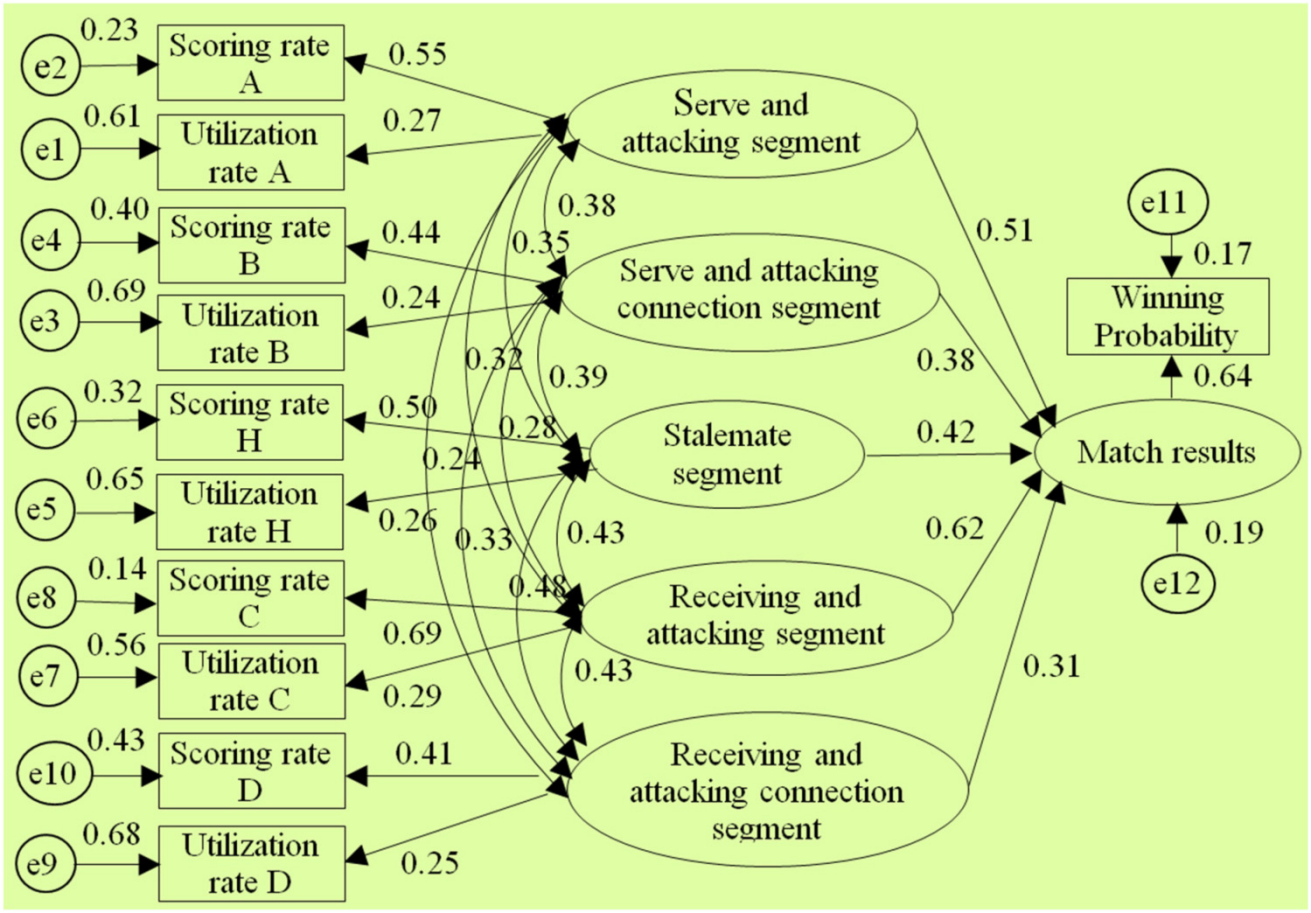
Diagram of the “Type⑧” diagnostic indicator system architecture model.

After using AMOS 17.0 to perform model estimation on the selected 7 segmented diagnostic index system structural models by conducting the “compute estimates” operation, it was found that only the structural models of the 1st, 2nd, 3rd, 5th, and 8th diagnostic index systems could be identified.(The test report is shown in Figures 3–7 and also shown in Tables 4 and 5), the structural models of the 6th and 9th diagnostic index systems could not be identified, indicating that these two structural models do not align with table tennis tactical data in the New Ball Era. Thus, the following text will conduct a quality inspection and comparison of five identifiable structural models.

**Table 4:** Each technique and tactics diagnostic Index architecture-fitness Index of structural model diagram.

Species	*χ*2	df	P	χ2/df	GFI	AGFI	NFI	RFI	CFI	RMSEA
①	36.83	9	0.267	4.09	0.938	0.907	0.896	0.871	0.896	0.071
②	39.62	9	0.291	4.41	0.915	0.897	0.872	0.852	0.907	0.085
③	71.37	9	0.085	7.93	0.784	0.739	0.813	0.764	0.739	0.125
⑤	101.81	18	0.157	5.66	0.864	0.861	0.866	0.798	0.887	0.111
⑧	81.34	30	0.488	2.71	0.937	0.909	0.914	0.896	0.918	0.039

**Table 5:** Standardized regression coefficient and significance test of each structural model.

Category	Species	Path	Estimate	S.E.	C.R.	P
Three-stage	①	Match outcome←serving-attacking segment	0.492	0.028	4.751	***
		Match outcome←receiving-attacking segment	0.614	0.073	7.527	***
		Match outcome←sustaining segment	0.652	0.051	5.474	***
	②	Match outcome←serving-attacking segment	0.653	0.086	2.515	0.006
		Match outcome←receiving-attacking segment	0.609	0.031	3.642	***
		Match outcome←sustaining segment	0.527	0.047	3.753	***
	③	Match outcome←serving-attacking segment	0.653	0.068	2.983	0.004
		Match outcome←receiving-attacking segment	0.567	0.033	2.419	0.007
		Match outcome←sustaining segment	0.456	0.052	4.228	***
Four-stage	⑤	Match outcome←serving-attacking segment	0.513	0.035	4.826	***
		Match outcome←receiving-attacking segment	0.623	0.028	7.835	***
		Match outcome←serving rotation sustaining segment	0.489	0.109	2.357	0.009
		Match outcome←receiving rotation sustaining segment	0.408	0.044	2.244	0.011
Five-stage	⑧	Match outcome←serving-attacking segment	0.508	0.037	3.541	***
		Match outcome←receiving-attacking segment	0.624	0.071	3.758	***
		Match outcome←serving-attacking connection segment	0.382	0.053	4.432	***
		Match outcome←receiving-attacking connection segment	0.310	0.074	4.643	***
		Match outcome←sustaining segment	0.423	0.049	5.822	***

Note: The *** symbol in the P column denotes that the *p*-value is less than 0.001, which indicates a very strong statistical significance.

#### 3.2.4 Quality inspection and comparison of the model

In a study (43), the author believes that the quality inspection of structural models is divided into two parts, the evaluation of external quality and the examination of internal quality. In the assessment of external quality (i.e., overall model fit), a *χ*2/df ratio less than 5 is considered acceptable, and less than 2 is good; GFI, IFI, NFI, CFI, and RFI should be greater than 0.9, with higher values indicating better fit; RMSEA should be less than 0.08, and lower values indicate better fit; In the examination of internal quality, the focus is primarily on understanding the reliability, validity of latent constructs, and whether the causal relationships defined by the theoretical constructs are valid.

According to the above test report, in the evaluation of external quality of the models (i.e., overall fit), there were no significant differences between the observed sample data and various structural models for the 1st, 2nd, 3rd, 5th, and 8th models (all *χ*2 significance probabilities were greater than 0.05), indicating that all structural models can be accepted. This also means that the five diagnostic indicator systems have a certain extent of rationality and can reflect to some extent the table tennis technical and tactical performance in the New Ball Era. Then, according to the standards for the external quality inspection of structural models, a comprehensive analysis of fit indicators such as *χ*2/df, NFI, GFI, RFI, AGFI, CFI, IFI, and RMSEA revealed the overall goodness-of-fit of the five structural models, from highest to lowest, is as follows: “Type⑧”, “Type①”, “Type②”, “Type⑤”, “Type③”, indicating the diagnostic indicator system “Type⑧” is the most optimal, better aligning with the table tennis tactical performance in the New Ball Era, while the diagnostic indicator system “Type③” has the lowest level of alignment.

Meanwhile, through comparing the goodness-of-fit results of the structural models of the aforementioned five diagnostic indicator systems and further analysis of their segment structure composition, it was found: Firstly, the goodness-of-fit of the structural models will be relatively better when the serving and attacking segment includes the 1st and 3rd strokes, and the receiving and attacking segment includes the 2nd and 4th strokes. Secondly, when the 5th stroke and the 6th stroke are respectively classified into the serving and attacking segments as well as receiving and attacking segment, and then the sustaining segment after the 6th stroke is divided into two parts, the goodness-of-fit of the structural models will gradually deteriorate. This suggests that in the New Ball Era, firstly, the composition structure of the serving and attacking segment as well as the receiving and attacking segment should still be maintained with the classic three-stage board configuration. Second, the classification of segments on the 5th stroke and 6th stroke should be carefully defined. Third, it is again empirically demonstrated that the sustaining segment after the 6th stroke should not be undefinedly divided into two parts. Thus, this also to some extent reveals the reasons for the differences in goodness-of-fit among the structural models and the optimal nature of the “Type⑧” structural model.

In the test of the intrinsic quality of the model (i.e., intrinsic structural fitness), the parameter estimates (C.R.) of the latent variables of the five structural models were greater than 1.96, indicating that the indicator variables measured (i.e., scoring rate and utilization rate) were effective in reflecting the latent variables (i.e., segments) that it was intended to measure, this further proves that these 5 structural models all have a certain degree of rationality. In addition, since the path coefficients between variables are important parameters reflecting the relative importance of the paths and the explanatory power of the independent variables on the dependent variable (43), to further reveal the extent to which each independent variable in the structural model affects the competition results, further analysis was conducted on the parameter estimation values of each structural model, especially the optimal structural model. The results showed:

Firstly, in terms of the importance of the “attacking” segment, the “connecting” segment, and the “sustaining” segment on the competition results, in the optimal structural model, the sum of the path coefficients for the serving-attacking segment as well as the receiving-attacking segment is 1.113, the highest; The sum of the path coefficients for the serving-attacking connection segment and the receiving-attacking connection segment is 0.69, ranking the second; the path coefficient for the sustaining segment is 0.42, the lowest. This indicates that the “attacking” segment has the greatest impact on the competition results, followed by the “connecting” segment, and the “sustaining” segment has the smallest impact. In addition, the correlation coefficient between the serving-attacking segment and the sustaining segment is 0.35, which is lower than the correlation coefficients of 0.38 and 0.39 between it and the serving-attacking connection segment, as well as the serving-attacking connection segment and the sustaining segment; The correlation coefficient between the receiving-attacking segment and the sustaining segment is 0.34, which is lower than the correlation coefficients of 0.43 and 0.48 between it and the receiving-attacking connection segment, as well as the receiving-attacking connection segment and the sustaining segment, respectively. This indicates that the position of the “connecting” segment in the New Ball Era is highlighted, playing a crucial role as a bridge between the “attacking” and “sustaining” segments. This closely corresponds to the fundamental logic of “the pavilion closest to the water enjoys the moon first” when woven conflicts occur from one board to another during matches, which further demonstrates the optimality of the 8th diagnostic indicator system.

Secondly, in terms of the importance of each “attacking” segment to the match outcome, in three structural models such as the 8th, 1st, and 5th structural model(where the serving-attacking segment includes the 1st and 3rd stroke, and the receiving-attacking segment includes the 2nd and 4th stroke), the path coefficients between the serving-attacking segment and the match outcome are 0.51, 0.49, and 0.51 respectively, all of which are less than the path coefficients between the receiving-attacking segment and the match outcome, which are 0.62, 0.61, and 0.62. This indicates that the receiving-attacking segment has a greater impact on the match outcome than the serving-attacking segment. Meanwhile, in each structural model, the correlation coefficients between the receiving-attacking segment and each of the other segments are generally higher than the correlation coefficients between the serving-attacking segment and other segments. It suggests that the receiving-attacking segment has a higher combined indirect effect on the outcome of the competition than the serving-attacking segment by organically linking and influencing the other segments.

Thirdly, in terms of the importance of each “connecting” segment to the match outcome, in the optimal structural model, the path coefficient between the serving-attacking connection segment and the match outcome is 0.38, which is higher than the path coefficient of 0.31 between the receiving-attacking connection segment and the match outcome. This indicates that the serving-attacking connection segment has a greater impact on the match outcome than the receiving-attacking connection segment.

## 4 Discussions

### 4.1 Comparative analysis of the scientific of diagnostic Index systems

The structural equation model analysis indicates that the “Type⑧” five-stage diagnostic index system is optimal and best fits the performance of table tennis tactics in the New Ball Era. The five stages are the serving-attacking segment, receiving-attacking segment, serving-attacking connection segment, receiving-attacking connection segment, as well as the sustaining segment. Among them, the serving-attacking segment includes the 1st and 3rd strokes, the receiving-attacking segment includes the 2nd and 4th strokes, the serving-attacking connection segment includes the 5th stroke, the receiving-attacking connection segment includes the 6th stroke, and the sustaining segment includes all strokes after the 6th stroke. As the creator of the five-stage diagnostic index system, Jiang Jinjun (20) based the construction of this index system on three factors. Firstly, the overall utilization rate of the 5th and 6th strokes in recent matches has significantly increased, the overall utilization rate of the “sustaining segment” after the sixth stroke is roughly equivalent. Additionally, the 5th and 6th strokes are pivotal turning points for the offense and defence in the competition; Secondly, the transition of the statistics directly from the serving-attacking segment, receiving-attacking segment to the sustaining segment is not conducive to reflecting the important impact of the first 2 segments on the sustaining segment; Thirdly, the strokes after the 5th and 6th stroke have relatively longer distances between serving and receiving, thus the influences received are relatively small, more with a real sense of the sustaining characteristics (20).Therefore, it is necessary and reasonable to separately classify the 5th and 6th strokes from the original sustaining segment for statistical analysis. In fact, the Chinese table tennis community recognized the importance of the 5th and 6th strokes at an early stage. As Li Xiaodong (44) conducted research on the connection technique of the 4th and 6th strokes after Primoratz's receiving-serving attack (44); Zhou X. pointed out that the connection between the 3rd and 5th strokes in the serving-attacking rotation and the connection between the 4th and 6th strokes after receiving-serving have become indispensable elements in the table tennis philosophy (45). Following the implementation of the new ball, the author's sequential empirical research (2018, 2019) further indicated that in the New Ball Era, the application and scoring characteristics of table tennis technics and tactics exhibit a three-stage pattern in the 1st to 4th strokes, the 5th to 6th strokes, the 7th stroke and subsequent strokes. Among them, the characteristics of the fifth and sixth strokes are significantly different from the “attacking” characteristics in the 1st to 4th strokes, as well as the true “sustaining” characteristics after the sixth stroke, instead, it demonstrates a “connecting” feature from “attacking” to “sustaining”; Moreover, the technical and tactical performance characteristics of the 5th and 6th strokes also differ (13, 22, 40, 46). Zhou Z.'s study also pointed out that the 5th and 6th strokes are key points of transition between offense and defense, playing a role in reversing the situation (19). From all the above, compared to the three-stage and four-stage as well as the six-stage diagnostic index systems, the new five-stage diagnostic index system developed on the basis of the classic three-stage system better fits the current stage of table tennis technical and tactical performance. It achieves a better inheritance and development of the classic three-stage method, and it is the most representative, scientific, and leading diagnostic index system in the New Ball Era.

Next, structural equation modeling analysis also indicated that the overall fit of the “Type③” structural model is the lowest, suggesting that the 5th and 6th strokes are not suitable for simple categorization into serving-attacking segment and receiving-attacking segment. The main reason is that, for men's table tennis matches, The “attacking” segment of the first stroke to the fourth stroke and the “connecting” segment of the fifth and sixth stroke exhibit different technical and tactical characteristics. Additionally, the tactical utilization of the 1st to 6th strokes, is relatively complex and diverse (13, 26, 27, 40). If the 5th stroke and 6th stroke are not distinguished and are simply categorized into respectively serving and attacking segment and receiving-attacking segment, it would fail to highlight the core winning function of serving and attacking on the 1st and 3rd strokes, and receiving and attacking on the 2nd and 4th strokes, it also does not help to demonstrate the important role of the connection between the 3rd stroke and the 5th stroke, and the 4th stroke as well as the 6th stroke as key turning points in the transition between offense and defense during the competition. As a result, this has led to the lowest fit and scientific validity of diagnostic indicator system “Type③” in the empirical comparison of the five diagnostic indicator systems that can be identified.

Additionally, the structural equation modeling analysis also indicates that the failure to identify the two diagnostic indicator systems “Type⑥” and “Type⑨” may be related to, on one hand, the rigid segmentation of the 5th stroke leading to discrepancies between statistical data and actual game situation, and on the other hand, it may also be related to the division of the sustaining segment after the 6th stroke into the serving rotation sustaining segment and the receiving and serving rotation sustaining segment. In fact, unless for specific research purposes, the sustaining segments after the 6th stroke are no longer suitable to be further divided into two parts, mainly due to: First, the scoring rates of the serving rotation sustaining segment and the receiving and serving rotation sustaining segment after the 6th stroke are quite close and difficult to distinguish (13, 22, 40, 47, 48); Secondly, from the perspective of the balanced utilization rate of each segment, the utilization rate of the 5th and 6th strokes is generally on par with the utilization rate of each stroke after the 6th stroke. If the sustaining segments after the 6th stroke are divided into two parts, the utilization rates of these two segments would be too small. This would not only disrupt the relative balance of utilization rates among segments but also diminish their reference value for winning matches.

### 4.2 Analysis of the impact of each segment on match outcomes

The relevant empirical research (13, 19, 22, 26, 49) comprehensively shows that in the New Ball Era, the technical and tactical application of the 1st to 4th strokes, the 5th to 6th strokes, and the 7th stroke as well as subsequent strokes respectively play the first, second, and 3rd important roles in determining the match outcome; In the 1st to 4th strokes, the impact of the receiving-attacking segment on the match result is greater than that of the serving-attacking segment. In the 5th to 6th strokes, the impact of the serving-attacking connection segment on the match result is higher than that of the receiving-attacking connection segment. The analysis of parameter estimation values of various structural models, especially the optimal five-stage structural model in this study, shows that firstly, the impact of the “attacking” segment on the match outcome is the greatest, followed by the “connecting” segment, and finally the “sustaining” segment has the smallest impact; Secondly, the impact of the receiving-attacking segment on the match result is greater than that of the serving-attacking segment; Thirdly, the impact of the serving-attacking connection segment on the match result is higher than that of the receiving-attacking connection segment, thereby verifying the research findings of previous studies. The prominent position of the 5th and 6th strokes is mainly due to two reasons: One reason is the rapid development and increasing importance of offensive receiving and serving skills and counter-attacking skills cantered around backhand loops, backhand rips, and backhand blocks, these have become critical factors in determining the outcome of competitions. which have become key factors in determining the outcome of matches, posing great challenges to the attacking play on the 2nd, 3rd, and 4th strokes. To better overcome the opponent's receiving and counter-looping, and to reduce the passive or erroneous situations caused by forced attacking or being counter-looped after attacking, strengthening the connection between the 3rd and 5th strokes, as well as the 4th and 6th strokes, and appropriately shifting the scoring emphasis from the “attacking” segment to the “connecting” segment, has become a necessary strategic choice (16, 22, 50, 51); Secondly, research has shown that “quick connection” has evolved into a highly threatening winning paradigm due to its characteristics of speed, tight connection, strong explosiveness, and significant pressure (11, 27, 44, 52, 53), which to a certain extent validates the importance of the 5th and 6th strokes.

## 5 Conclusion and recommendations

### 5.1 Conclusion

In the New Ball Era, each segmented diagnostic index system has a certain degree of reasonableness, but in comparison, the five-stage diagnostic index system is optimal and best fits the technical and tactical performance in the New Ball Era.

The five-stage diagnostic index system includes the serving-attacking segment, receiving-attacking segment, serving-attacking connection segment, receiving- attacking connection segment, and the sustaining segment. The serving-attacking segment consists of the 1st and 3rd strokes, the receiving-attacking segment consists of the 2nd and 4th strokes, the serving-attacking connection segment and the receiving-attacking connection segment consists of the 5th and 6th strokes respectively, and the sustaining segment consists of the strokes after the 6th stroke. Among them, the “attacking” segment has the greatest impact on the outcome of the competition, the “connection” segment is secondary, and the “sustaining” segment has the least impact. The receiving-attacking segment has a greater impact on the outcome of the competition than the serving-attacking segment, and the serving-attacking connection segment has a greater impact on the outcome of the competition than the receiving-attacking connection segment.

In the New Ball Era, the “connection” segment of the 5th and 6th strokes takes on a prominent position, play a pivotal role in transitioning in both attack and defense between the “attacking” segment and the “sustaining” segment.

### 5.2 Recommendation

Based on the fact that the five-stage diagnostic index system in the New Ball Era is the most suitable for the technical and tactical performance of table tennis, it is recommended that the five-stage diagnostic index system be adopted when carrying out the diagnostic analyses of men's technique and tactics, in order to maximize the effectiveness of technical and tactical diagnosis.

In training, it is recommended to treat the “attacking” segment, the “connecting” segment, and the “sustaining” segment differently according to their relative importance to match results. Secondly, in view of the new features that highlight the importance of the connection segment between the 5th and 6th strokes in the New Ball Era, it is essential to enhance targeted training on the connecting ability of the 5th and 6th strokes. On the one hand, efforts should be made to advance the technical and tactical capabilities of the 1st to 4th strokes in a deeper direction. On the other hand, it is essential to lay the groundwork and create conditions for proactively entering a sustaining segment.

## 6 Research prospects

With the continuous advancement of table tennis techniques, ongoing modifications to the rules, and innovations in equipment, the technical and tactical aspects of the sport are inevitably evolving. Consequently, on one hand, the diagnostic index systems for techniques and tactics require corresponding adjustments and reconstruction. On the other hand, research into these diagnostic index systems should extend beyond purely theoretical explorations or small-scale studies; instead, it necessitates the construction of such systems based on large-sample empirical research.

## Data Availability

The original contributions presented in the study are included in the article/Supplementary Material, further inquiries can be directed to the corresponding author/s.
